# A Comparison of the Dynamics of S100B, S100A1, and S100A6 mRNA Expression in Hippocampal CA1 Area of Rats during Long-Term Potentiation and after Low-Frequency Stimulation

**DOI:** 10.1155/2010/720958

**Published:** 2010-08-30

**Authors:** Pavel D. Lisachev, Mark B. Shtark, Olga O. Sokolova, Vladimir O. Pustylnyak, Mary Yu. Salakhutdinova, Oleg I. Epstein

**Affiliations:** ^1^Institute for Molecular Biology and Biophysics, Siberian Branch of the Russian Academy of Medical Sciences, 2 Timakova Street, Novosibirsk 630117, Russia; ^2^Laboratory of Biomedical Informatics, Design Technological Institute of Digital Techniques, Siberian Branch of The Russian Academy of Sciences, 6 Institutskaya ul., Novosibirsk 630090, Russia; ^3^Research Division, OOO NPF Materia Medica Holding, 9 3rd Samotyochnyi per., Moscow 127473, Russia

## Abstract

The interest in tissue- and cell-specific S100 proteins physiological roles in the brain remains high. However, necessary experimental data for the assessment of their dynamics in one of the most important brain activities, its plasticity, is not sufficient. We studied the expression of S100B, S100A1, and S100A6 mRNA in the subfield CA1 of rat hippocampal slices after tetanic and low-frequency stimulation by real-time PCR. Within 30 min after tetanization, a 2–4 fold increase of the S100B mRNA level was observed as compared to the control (intact slices) or to low-frequency stimulation. Subsequently, the S100B mRNA content gradually returned to baseline. The amount of S100A1 mRNA gradually increased during first hour and maintained at the achieved level in the course of second hour after tetanization. The level of S100A6 mRNA did not change following tetanization or low-frequency stimulation.

## 1. Introduction

Although the S100 proteins are known to have a broad spectrum of intra- and extracellular functions, the roles they play in the central nervous system, in general, and, particularly, in mediating one of the most important brain activities, its plasticity, are largely unknown. Most S100 proteins undergo conformational changes upon calcium binding, which allows them to interact with target proteins [1]. They are differentially expressed in a variety of cell types and tissues, and are thought to play unique roles, despite a high degree of sequence homology they share and a significant overlap of their expression patterns. For example, in the brain, S100A1 is predominantly expressed in neurons, S100B expression maps to the astrocytes of hippocampus and cortex, as well as to the subpopulations of oligodendrocytes and neurons, while S100A6 is mainly detected in neurons of the restricted brain areas (amygdala and entorhinal cortex) and in some astrocytes [2–5]. At least several S100 proteins affect cell growth, for example, S100B and S100A1, which interact with the tumor suppressor p53 [6, 7].

Among S100 proteins, S100B is the most studied one in connection with the neuronal plasticity, although the data available is contradictory. Antiserum against S-100 protein prevents the long-term potentiation (LTP) in the CA1 region of rat hippocampal slices, suggesting a positive role of S100B in the manifestation of LTP [8]. In contrast to this observation, another set of experiments indicate at a rather negative influence of S100B on LTP [9, 10]. Transgenic mice overexpressing the human S100B protein exhibited the impaired hippocampal LTP and spatial learning [9]. Conversely, an enhanced LTP was observed in the hippocampal CA1 region of mutant mice devoid of S100B, while the perfusion of hippocampal slices with the recombinant S100B protein reversed the LTP in the slices from mutant mice to the wild-type level [10]. The expression of both S100A6 and S100B proteins has been shown to be modulated in the course of human brain development [11]. Higher levels of S100B have been detected in the sera of patients after brain trauma or ischemia, as well as those suffering from Alzheimer disease or Down syndrome [12]. Overexpression of S100A6 has also been observed in patients suffering from Alzheimer disease or amyotrophic lateral sclerosis [13, 14]. S100A1 plays a certain role in modulating innate fear and exploration of novel stimuli [15].

The long-term brain plasticity requires the changes in gene expression [16, 17]. Complex spatiotemporal patterns of the expression of new growth factors, ion channels, structural molecules, and other proteins specify the alterations of neuronal circuitry. With the purpose of studying the role of gene expression in mediating the mechanisms of neuronal plasticity, we compared the dynamics of mRNA expression for three highly homologous S100 proteins with different brain localizations after the LTP-inducing tetanization or the low-frequency stimulation (LFS), which did not induce LTP, in hippocampal CA1 area of rats.

## 2. Materials and Methods

Experiments were conducted on hippocampal slices prepared routinely. Wistar male rats (weight 180–220 g) were decapitated, the brains were rapidly removed and placed into ice-cold oxygenated (95% O_2_, 5% CO_2_) artificial cerebrospinal fluid (aCSF): 126 mM NaCl, 4 mM KCl, 1.24 mM NaH_2_PO_4_, 1.3 mM MgSO_4_, 2.3 mM CaCl_2_, 26 mM NaHCO_3_, 10 mM D-glucose, pH 7.4. Left hippocampus was dissected for removal and cut into 400 *μ*m-thick slices perpendicular to the longitudinal axis, using a chopper. Four consecutive slices from dorsolateral region of the left hippocampus were transferred to a submerged recording chamber (volume 7 mL). Slices were continuously perfused with the fresh, oxygenated aCSF warmed to 32–33°C at a rate of 2 mL/min. For extracellular recordings, the recording electrode filled with aCSF (resistance 1–5 M*Ω*) was placed in the hippocampal CA1 pyramidal cell layer. To stimulate Schaffer's collaterals, a bipolar electrode with a tip diameter of 50 *μ*m filled with aCSF was placed in the CA1 stratum radiatum. Intensity of stimulation was adjusted to get p-spike amplitude ~50% of the maximal response.

Two separate series of experiments were carried out: the first, to test efficiency of the stimulation protocols in our hands, and the second, to obtain the samples for real time PCR analysis. While testing the stimulation protocol efficiency, a single slice from each animal was used. Tetanization (4 series × 1 s, 100 Hz, with 30 s intervals) or low-frequency stimulation (400 stimuli during 91 s, i.e., at 4.4 Hz) were performed 2.5 hr postdissection. Control slices were not treated by tetanization or LFS protocols and received only test stimulation in these experiments.

**Figure 1:**
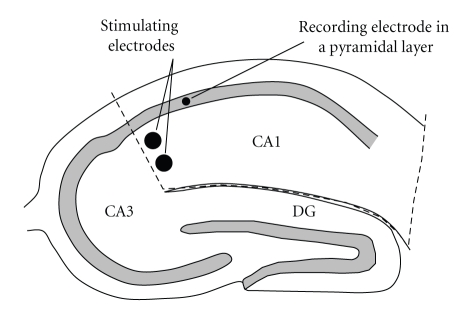
The region of the hippocampal slice (CA1) used for mRNA sample preparation. CA1, CA3, DG—hippocampus subfields. Hatched line indicates the direction of sections made to separate the studied region.

Sample preparation for real time PCR analysis was as follows. The slice, which was used as a baseline control for the mRNA content normalization remained intact throughout the entire incubation period (4.5 hrs). Three other slices were tetanized (or subjected to LFS) 120, 60, or 30 min prior to the end of incubation. Electrodes were placed onto the slices 10 min before and were removed immediately after the stimulation. At the end of incubation, slices were transferred to an ice-cold oxygenated aCSF, the CA1 field was rapidly dissected from each slice (as shown in Figure 1) and placed into liquid nitrogen. Five relevant slices from different animals were pooled to prepare each mRNA sample.

Total RNA was isolated from frozen slices using Aurum Total RNA Fatty and Fibrous Tissue Kit (Bio-Rad Laboratories) according to the manufacturer's protocol. The RNA concentration and purity were determined spectrophotometrically by measuring the absorbance at 260 and 280 nm (corrected for background at 320 nm), and the RNA integrity was assessed by running the sample on a denaturing agarose (1%) gel and visualization of 18S and 28S rRNA bands. As much as 0.5 *μ*g of total RNA was used for single-stranded cDNA synthesis. First strand cDNA synthesis was carried out using iScript cDNA Synthesis Kit (Bio-Rad Laboratories) according to the manufacturer's protocol. Gene expression levels were assayed by real-time PCR using iTaq SYBR Green Supermix with ROX (Bio-Rad Laboratories). Real-time PCR experiments were performed with IQ5 Real-Time PCR instrument (Bio-Rad Laboratories). The “house-keeping” gene, *β*-actin, was used as an endogenous internal control for normalization. The gene-specific primer sequences for S100B, S100A1, S100A6, and *β*-actin amplification were as follows: S100B F: 5′-TTGCCCTCATTGATGTCTTCCA-3′, R: 5′-TCTGCCTTGATTCTTACAGGTGAC-3′; S100A1 F: 5′-CCATGGAGACCCTCATCAAT-3′, R: 5′-TTCTGGACATCCAGGAAGC-3′; S100A6 F: 5′-CTTCTCGTGGCTATCTTCC-3′, R: 5′-ACTGGACTTGACTGGGATAG-3′; *β*-actin F: 5′-ACCCACACTGTGCCCATCTA-3′, R: 5′-CGGAACCGCTCATTGCC-3′.

The optimal annealing temperature for each primer set was determined prior to the experimental sample analysis. Duplicate real-time PCR reactions were run for each sample containing SYBR Green master mix, 300 nM forward and reverse primers, and 25 ng cDNA template in 25 *μ*L of a total reaction volume. The following standard real-time PCR conditions were used: one cycle of 95°C for 3 min and 40 cycles of 95°C for 15 s, the primer specific annealing temperature (58°C) for 20 s, 72°C for 20 s, optical data were collected at 80°C for 10 s. After PCR experiments, the dissociation curve was established using the built-in melting curve program to confirm the presence of a single PCR product, which was then confirmed by gel electrophoresis. The fold change in the target gene, normalized to *β*-actin and relative to control, was calculated based on PCR efficiency (E) and Ct. 

Data is expressed as the mean ± S.E.M and tested for statistical significance using Student's *t*-test (for the comparison of the amplitudes of p-spikes after tetanization or LFS with the baseline), paired *t*-test (for the comparison of mRNA amounts in the samples from stimulated and control/nonstimulated slices), and repeated measures ANOVA (for the comparison of mRNA dynamics after tetanization and LFS).

## 3. Results

**Figure 2:**
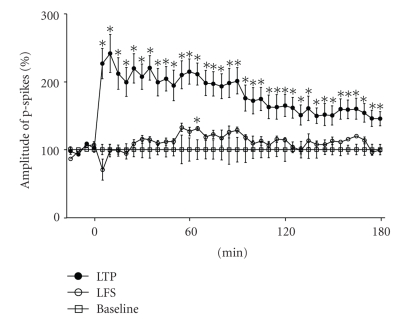
The influence of tetanization and low-frequency stimulation on the amplitude of p-spikes in pyramidal layer of CA1. P-spikes were elicited by stimulation of Shaffer's collaterals. Abscissa, the time from the onset of tetanization (LTP, long-term potentiation) or low-frequency stimulation (LFS). Ordinate, the amplitude of p-spike, first, normalized to the average amplitude of p-spikes in 4 responses prior to tetanization (*n* = 5) or LFS (*n* = 3), and then, to the mean amplitude of responses in the control group (baseline, *n* = 4). *P_*t*_ < .05, against baseline, Student's *t*-test.

As shown in Figure 2, the employed tetanization protocol caused a significant increase in the responses in CA1 field to the stimulation of Schaffer's collaterals. The response level remained increased throughout the entire course of observation (3 hrs). The same amount of 4.4 Hz stimuli (LFS) applied within the same time frame did not cause any potentiation.

**Figure 3:**
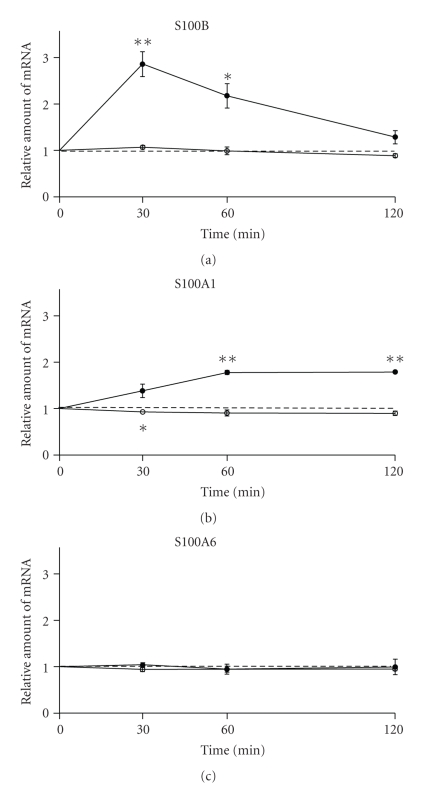
The dynamics of S100B, S100A1, and S100A6 relative mRNA amounts in hippocampal CA1 area of rats after tetanization and low-frequency stimulation. Ordinate, relative amount of mRNA normalized to relative amount of mRNA in control samples (nonstimulated slices). Abscissa, time after stimulation (min). S100B: *n*1 = 4, *n*2 = 3; S100A1: *n*1 = 3, *n*2 = 3; S100A6: *n*1 = 4, *n*2 = 3 for tetanization (black circles) and low-frequency stimulation (white circles), respectively. **P_*t*_ < .01, *P_*t*_ < .05, against control samples, Student's paired *t*-test.

Tetanization increased the S100B mRNA level drastically and rapidly (Figure 3). As early as 30 min after tetanization, a substantial increase of S100B mRNA level was observed (286 ± 27%, *P*_*t*_ = .006, *n* = 4) in treated specimens, as compared to the control (nonstimulated) slices. Then S100B mRNA level gradually returned to baseline (218 ± 26% at 60 min, *P*_*t*_ = .02, *n* = 4) and, within 120 min after tetanization, it was not differing from the control values (128 ± 14%, *P*_*t*_ = .14, *n* = 4). LFS did not cause any changes of the S100B mRNA level at studied time points (Figure 3). 2-Way repeated measures ANOVA (group(2) × time(3)) showed highly significant difference in the amount and dynamics of S100B mRNA between tetanization and LFS (group effect F_(1,5)_ = 21, *P* < .006; group-time interaction F_(2,10)_ = 19, *P* < .001).

The S100A1 mRNA content, conversely, increased slowly (during the first hour) and was maintained at the achieved level in the course of the second hour after tetanization (Figure 3) (30 min, 138 ± 30%, *P*_*t*_ = .15, *n* = 3; 60 min, 177 ± 10%, *P*_*t*_ = .004, *n* = 3; 120 min, 179 ± 1%, *P*_*t*_ ≪ .001, *n* = 3). 2-Way repeated measures ANOVA (group(2) × time(3)) showed highly significant difference in the amount of S100A1 mRNA between tetanization and LFS (group effect F_(1,4)_ = 314, *P* ≪ .001; group-time interaction F_(2,8)_ = 3.7, *P* = .07).

Analysis of the S100A6 mRNA content did not reveal any difference between treated and control samples at all time points studied both after tetanization and following LFS (Figure 3). Correspondingly, 2-Way repeated measures ANOVA (group(2) × time(3)) showed insignificant differences between tetanization and LFS in respect to the amount or dynamics of S100A6 mRNA after stimulation (group effect F_(1,5)_ = 0.4, *P* = .56; group-time interaction F_(2,10)_ = 0.1, *P* = .89).

## 4. Discussion

Gene transcription is required for establishing and maintaining the enduring form of long-term potentiation. The products of coordinated expression of a multitude of genes enable the stable modification of synaptic efficiency and neuronal excitability [18, 19]. In the past years, the classical view that astrocytes play a relatively passive role in brain function has been overturned, and it has become increasingly clear that signaling between neurons and astrocytes may play a crucial role in the information processing performed by the brain [20]. Investigation and characterization of not only neuronal, but also the glial transcriptional profiles after tetanic stimulation will provide a better understanding of the processes underlying the long-term changes in response to neuroglial activation.

We showed that LTP-inducing tetanization of Schaffer's collaterals, but not LFS, evokes increases in S100B and S100A1 mRNA levels in hippocampal CA1 area of rats. This result suggests that tetanization-induced changes in S100B and S100A1 mRNA amounts were not associated with mechanical or electric damage of slices with electrodes, but might rather be relevant to the processes underlying neuronal plasticity. The expression of S100B in astrocytes is upregulated by brain-derived neurotrophic factor (BDNF) [21], which is thought to play a crucial role in LTP mechanisms [22]. BDNF activity during LTP initiation might possibly participate in the induction of a rapid and transient increase of S100B mRNA content after tetanization, although a number of other mechanisms of S100B upregulation exist [23], including the spillover of glutamate from activated synapses. Extracellular S100B evokes an increase in intracellular free calcium concentration in glial and neuronal cells [24]. Calcium is involved in a number of cellular processes including the regulation of transcription. It is, therefore, possible that increased S100B protein secretion from astrocytes associated with neuronal activation [25] might induce S100B gene transcription.

In contrast to our results, LTP induction in mouse dentate gyrus slightly decreased S100B mRNA content [19], however, the reasons for that discrepancy remain unclear. It would be interesting to compare the baseline expression of S100B mRNA in our control (nonstimulated slices) samples (35 ± 5% of the *β*-actin mRNA, *n* = 7) with those in mice. Chang et al. [19] used minislices of dentate gyrus dissected from the whole hippocampal slices immediately after their preparation, while we worked on whole slices and dissected CA1 immediately before freezing. Supposedly, a more extensive injury to the slices, as in [19], might be responsible for a more prominent basal expression of S100B and, thereby, might occlude its further increase after LTP. 

Functional significance of the S100B mRNA increase after tetanization in our experiments is not yet clear. It would be worthwhile to investigate whether S100B protein content increases along with its mRNA expression. High frequency neuronal activation stimulates S100B secretion in hippocampal slices [25]. Whether the enhanced mRNA expression simply replenishes the pool of S100B protein or a further increase of the protein above baseline takes place remains to be elucidated.

As mentioned above, experimental data concerning the influence of S100B protein on LTP is contradictory. Natural sources of this discrepancy arising from the complexity of the biochemical machinery, which S100B is involved in, were discussed elsewhere [23]. Still another reason for contradictions could be the immanent instability of acute slices. Cutting hippocampal slices for in vitro experiments causes prolonged disturbance of the background genetic activity, which might never reach steady state. For example, the expression of interleukin-1*β* mRNA can increase steadily with the time over at least 7 hours after slice preparation [26]. Changing background genetic activity could influence the mechanisms of plasticity. A degree of LTP in hippocampal slices was reported to decrease during survival [27]. Unfortunately, this issue is commonly neglected by researchers. Thus, when a varying amount of slices from one animal is used in experiments and processed serially, it is difficult to exclude artifacts associated with the differences in times of slice survival in the compared experimental groups.

As most of the other S100 proteins, S100A1 is a multifunctional regulator. With respect to LTP mechanisms, an implication of S100A1 in the Ca^2+^-dependent regulation of synaptic vesicle trafficking and, ultimately, in the regulation of presynaptic function and plasticity [28] might be relevant. Though the data on brain S100A1 secretion is lacking so far, it is known to induce RAGE-dependent growth of neurites and promote neuronal survival in physiological concentrations [29]. Furthermore, it is shown that RAGE activation results in a fast CREB phosphorylation and its translocation into the nucleus [30], the factor being involved in LTP mechanisms [17]. 

We did not observe any changes in S100A6 mRNA following LTP induction. Obviously, this fact alone does not exclude S100A6 participation in LTP mechanisms, but, rather, reflects the peculiarities of the protein cellular distribution and/or expression regulation. As S100B, S100A1, and some other S100 proteins, S100A6 is a RAGE ligand and might be involved in mechanisms of cell survival [31].

Our results indicate that glial gene expression response to the neuronal activation can be quite appreciable, although functional significance of the observed increment in S100B mRNA content after tetanization remains to be clarified. We hope that further investigation of this phenomenon will extend our knowledge about neuroglial cooperation during LTP formation.
